# Exploring food security as a multidimensional topic: twenty years of scientific publications and recent developments

**DOI:** 10.1007/s11135-022-01452-3

**Published:** 2022-08-09

**Authors:** Maria Stella Righettini, Elisa Bordin

**Affiliations:** 1 Department of Political Science, Law and International Studies (SPGI), University of Padova, Milan, Italy; 2 Department of Social and Political Sciences, University of Milan, Milan, Italy

**Keywords:** Food security, Food policy, Text mining, Food poverty, Food access, Literature review

## Abstract

The scientific literature dealing with food security is vast and fragmented, making it difficult to understand the state of the art and potential development of scientific research on a central theme within sustainable development.

The current article, starting from some milestone publications during the 1980s and 1990s about food poverty and good nutrition programmes, sets out the quantitative and qualitative aspects of a vast scientific production that could generate future food security research. It offers an overview of the topics that characterize the theoretical and empirical dimensions of food security, maps the state of the art, and highlights trends in publications’ ascending and descending themes. To this end the paper applies quantitative/qualitative methods to analyse more than 20,000 scientific articles published in Scopus between 2000 and 2020.

Evidence suggests the need to find more robust links between micro studies on food safety and nutrition poverty and macro changes in food security, such as the impact of climate change on agricultural production and global food crises. However, the potential inherent in the extensive and multidisciplinary research on food safety encounters limitations, particularly the difficulty of theoretically and empirically connecting the global and regional dimensions of change (crisis) with meso (policy) and micro (individual behaviour) dimensions.

## Introduction

Food insecurity is a timely and multidimensional problem positioned at the crossroads between the right to food and health in developing and rich, industrialized countries. However, it is unclear how scientific production reflects this multidimensionality overall and whether the recent COVID-19 pandemic has shed new light on the issues at stake. The analysis presented in this article aims to give a systematic review and meta-analysis of the vast amount of scientific food security research produced over the past twenty years. This work aims to offer an overview of the topics that characterize food security’s theoretical and empirical dimensions, map state of the art, and highlight trends in scientific publications’ ascending and descending themes. A systematic literature review sets out quantitative and qualitative aspects which could generate future food security research.

Since the 1960s, with the approval of precursor USA federal anti-poverty programs (Esobi et al. 2021; Nestle 2019; Swann 2017), interest in preventing the adverse effects of poverty has broadened and deepened scientific interest in the field of how to guarantee food access to the neediest people. Since then, various framings, reflecting differences in meaning and problem formulation and coming from different territorial and disciplinary perspectives, have highlighted the contested relationship between social, economic, and environmental circumstances to food access and nutrition experiences (Dowler and O’Connor 2012). In the 1960s, creating the World Food Program (WFP) was a prominent example of the institutionalization of the ‘food for development’ framework. The food crisis of 1972–74 marked a turning point in food security insurance schemes and led to better coordination between donor countries. Then, the first official mention of food security was in the United Nations report presented at the World Food Conference in 1974 (McKeon 2014). In the 1980s and 1990s, food security was broadened to include physical and economic access to food and consider women’s role in poverty alleviation. Therefore, what has come to be termed food security and nutrition security has been controversial, reflecting multiple, not always coordinated, governmental policies and multifaceted theoretical and research fields.

The most used definition of food security was developed during the 1996 World Food Summit organized by the Food and Agriculture Organization (FAO) (Mechlem 2004). It resembles the definition of the right to food (Maxwell and Smith 1992; Smith et al. 1993). Food security exists when all people, at all times, have physical and economic access to sufficient, safe, and nutritious food to meet their dietary needs and food preferences for an active and healthy life (FAO 2003). Food security has become a priority focus for donor states and cooperation with developing countries to reduce poverty and systemic environmental, economic, and social causes of hunger. It has five conceptual dimensions: nutritional status, utilization, accessibility, availability, and stability (Gross et al. 2000). However, unlike food security, nutrition security refers only to the individual’s (mal)nutritional status due to diet regime, food intake, and health status (Gross et al. 2000).

Academic research developed many approaches to food insecurity during the 1980s and 1990s. The right-to-food-based approach to food security suggests that human dignity, rights acknowledgment, transparency, government accountability, citizens’ empowerment, food, and wellbeing should be considered in welfare programs. The right-to-food approach required governments to adopt specific programs and meet precise obligations to combat poverty (Maxwell 1996). The right to food was not merely a means to achieve food security; rather, it was seen as a broader, more encompassing, and distinct objective. The seminal works of Amartya Sen on poverty during the 1980s (Sen 1980, 1981, 1982) raised controversy over nutritional norms and intensified the debate on the interrelations between food access and poverty reduction interventions. Food security is seen as an integral part of social security, understood as the “prevention by social means, of a deficient standard of living irrespectively of whether these are the results of chronic deprivation or temporary adversity” (Burgess and Stern 1991:4). We should point out that this debate regarded achieving food security in the poorest developing countries and the richest developed ones, where obesity and malnutrition among low-income people increased. The multifaceted nature of food security is entrenched in its measurement complexity (in terms of life expectancy or income). To understand the causes of deprivation and fragility associated with the lives of increasing portions of the population, food security scholars have striven to assess the validity of tools measuring food safety/insecurity and provide valid indications and suggestions to policymakers.

In the 2000 Plan for Action regarding food and diet in Europe, the World Health Organization (WHO) argued that nutrition security in the 21st century depends on production that meets dietary needs and enables equal access to appropriate food while controlling misleading promotional messages (Carlson et al. 1999). In addition, food prices, policies, and education can significantly reduce malnutrition risks (Wekerle 2004).

Even though rising poverty and hunger levels have been a concern for many countries, acknowledgment and quantification of hunger have been disputed and hindered by the lack of an accepted definition and measure of food security. Before food security can be measured, the potential target of the intervention must be identified.

The first food security measure based on household experiences at the individual level was developed in 1990 by Radimer and colleagues (1992) and based on a 12-item questionnaire. The ‘hunger index’ was developed through qualitative interviews with women from low-income households (Kendall, Olson, and Frongillo 1995). Since 1992, the literature has indicated that food security measurements may vary in their performance across different population groups and cultures and that good practice and policy instruments are difficult to transfer across different contexts (Kendall, Olson, and Frongillo 1995; Leyna et al. 2008; Radimer and Radimer 2002; Zerafati Shoae et al. 2007). One of the main problems in rolling out food security interventions is that it is not easy to identify the target (households below the poverty line) (Carlson et al. 1999). Although such identification usually lacks accuracy, generalized subsidies (food stamps) – on commodities consumed by both the rich and the poor – have often been an attractive option for policymakers (Besley and Kanbur 1988).

The more recent Food Insecurity Experience Scale (FIES), developed by the Food and Agriculture Organization (FAO), focuses on food consumption experience, living conditions, and individual contexts (Cafiero et al. 2018). It consists of eight dimensions regarding people’s access to adequate food, and it is based on various kinds of population surveys. Its global reference scale is based on results from the application of the FIES survey module in countries covered by the Gallup World Poll in 2014, 2015, and 2016. In addition, food insecurity prevalence rates allow comparison between different countries, and the FIES is designed to measure unobservable traits such as aptitude/intelligence, personality, and a broad range of social psychology- and health-related conditions (Cafiero et al. 2018).

The food security issue has gained greater cross-cutting relevance in academic and policy circles in connection to public health issues related to the economic and social crises raised by the COVID-19 pandemic (Ahn and Norwood 2020; Arouna et al. 2020; Béné 2020; Cable et al. 2021; Mishra and Rampal 2020; Moseley and Battersby 2020; O’Hara and Toussaint 2021). Economic and social stresses generated by the pandemic led to the formulation of renewed public interventions in response to food insecurity in developing and rich countries.

The literature review presented in the following sections aims to fill a gap in our knowledge of the vast amount of scientific food security research produced, its theoretical and research dimensions, and trends in the last twenty years. Furthermore, the fully electronic search intends to illustrate the main lines of scientific interest within the topic and indicate the most transversal issues and promising areas of scientific interaction. Therefore, this article is organized as follows.

Section 2 illustrates the research questions and methods adopted to build the dataset of articles addressing food security. Section 3 presents research results regarding publications over time and across scientific areas. Section 4 describes the most recurrent topics and clusters of issues addressed by the food security literature. Section 5 shows their inter-relations and evolution over time. Section 6 presents an in-depth analysis of the thematic cluster on domestic programs. Section 7 shows the impact of COVID-19 in the thematic focus of publications. Finally, Section 8 discusses the main findings and limits of the meta-analysis and evaluates the contribution of the food security literature to unlocking the future research potential of transboundary policies and governance.

## Research questions and methods

This article aims to answer the three main research questions: in a systematic literature review, what are the main thematic dimensions linked to the issue of food security? How do these dimensions evolve, and how do they relate? Furthermore, does the literature highlight new dimensions of the problem concerning the COVID-19 pandemic?

To achieve its scope, this article combines bibliographic analysis, semi-automatic content analysis, and topic detection to explore the literature on food security. Following research domain analysis (RDA), applied to all publications in a given research domain (Janssen 2007; Janssen et al. 2006; Janssen and Ostrom 2006), we describe multiple strands of literature, in particular interdisciplinary and inter-sectoral ones, to highlight dimensions and research agendas linked to the food security theme. An overview of the scholarly production and its evolution is essential to account for its achievements and gaps and identify the way forward.

There is not a comprehensive literature review on food security so far, but only partial reviews (Candel 2014; Chan et al. 2006; Haddad et al. 1996; Nosratabadi et al. 2020; Thompson et al. 2010). Therefore, our objective is to conduct an exploratory literature review investigating the multidisciplinary approaches to the issue. Thus, when choosing the most appropriate data source to construct the database of articles, the comprehensiveness of content coverage was the most important criterion to evaluate. Bibliographic databases (DBs) are the leading providers of publication metadata and bibliometric indicators (Pranckute 2021). Scopus and Web of Science (WoS) are widely acknowledged as the two most comprehensive DBs (see Pranckute 2021 for a comprehensive review of the studies). However, multiple studies confirmed that Scopus has a more comprehensive overall coverage than WoS. In addition, while the content of the two databases is generally overlapping, Scopus indexes a more significant number of unique sources not covered by WoS, though this variation differs across specific subject fields (Pranckute 2021). Hence, we deemed Scopus the most appropriate data source for our literature review given its greater comprehensiveness.

To conduct our bibliometric analysis, we started by compiling a list of words and concepts related to food security based on seminal works in the literature and the authors’ knowledge of the topic. The list included the following terms: food security, food insecurity, food aid, food poverty, nutrition quality, food solidarity, and food stamps. Subsequently, we compiled a list of possible combinations of these terms using Boolean operators, and we used them as search strings to explore the title, abstract, or keywords of publications within the Scopus database. The choice of “food security” (in quotation marks) as a keyword resulted from an iterative process that involved multiple searches using all the compiled combinations and discussions among the authors. As a result, this keyword returned the highest number of articles (over 20,000 results, as opposed to less than 10,000 results for other combinations). Moreover, the articles citing the term “food security” also covered all the other terms and combinations, while the reverse was not valid. Hence, we verified that the concept of “food security” is the most comprehensive and that it contains other relevant frames, such as food poverty, nutrition poverty, and, to a lesser extent, food safety.

Due to the database consistency and the marked increase in the number of articles per year, we decided to focus on literature published during the last two decades, thus analyzing the articles produced between 2000 and 2020. Furthermore, we decided to analyze separately the literature published between January and July 2021 (the current year) to avoid a misinterpretation of the results. The 2021 literature is relevant for identifying a variation in themes and focuses after the COVID-19 pandemic.

A first search using the selected keywords returned 34,931 documents. To narrow the analysis, we decided to focus the search on peer-reviewed articles written in English, as they constitute the core of the international literature. In addition, we cleaned the resulting database by removing articles with no abstract and duplicates. To select which duplicate would be kept in the database, we respected the following criteria: the most recent, the longest abstract, and correct formatting. The final database contained 21,574 articles. For the first fundamental analysis of the database, we used the automatic analyses provided by Scopus and complemented them with analyses made through Microsoft Excel. The aim was to list the academic journals, countries, and scientific areas of the articles published.

The second step of the analysis focused on the thematic dimensions covered in the food security literature. We performed a topic detection on the articles’ abstracts to achieve this aim, using automatic content analysis. Automatic and semi-automatic content analyses are evolving trends in the literature. These methods exploit algorithms and software to apply statistical analysis to textual data in electronic format (Sbalchiero 2018). They automate the process of data encoding and analysis, thus combining the advantage of timesaving with the possibility to investigate the main topics and issues discussed in the literature without any prior theoretical or analytical constraints (Sbalchiero and Eder 2020; Righettini and Lizzi 2021). However, while their application to the analysis of social media and political documents is growing, they remain marginal in literature reviews. Still, this kind of analysis allows researchers to overcome the biases involved in literature reviews when the authors select and analyze articles. In many cases, the selection criteria for the articles are not specified, so there is a risk that essential studies will be left out, and a self-reinforcing mechanism will be perpetuated around a limited number of articles. Conversely, the automatic and semi-automatic analysis considers the whole body of literature, thus allowing researchers to explore the variety of theoretical frameworks and methodologies that unravel undetected patterns.

Topic detection is a text mining technique that allows detecting these patterns in large document corpora and classifying them as recurring topics and themes. The “latent” topics within the corpus’ documents are unveiled through algorithms that use statistical modeling and programming language to analyze the correlation among terms, i.e., words or phrases (El-Taliawi et al. 2021). This study employs Reinert’s method (Reinert 1990, 2001), which uses the R-based software Iramuteq to analyze “the co-occurrences of words as they appear in portions of text, and thereby identify lexical worlds, or semantic classes” (Sbalchiero 2018, p. 202). This method automatically performs most of the operations required to prepare the corpora (lemmatization, spelling harmonization, etc.). It allows for saving time in the process and increasing precision. Moreover, it does not require specifying the number of topics a priori (Sbalchiero and Eder 2020). Thus, it appears more fitted for explorative analysis of the literature we aim to do in this study.

The algorithm implemented by Iramuteq constructs a contingency matrix of words-per-abstract based on the co-occurrences of words in each abstract. It then uses a clustering procedure that hierarchically identifies the “factors (clusters) that best represent a lexical world from the distance of the chi-square between the classes” (Sbalchiero 2018, p. 203). Pearson’s chi-square test (statistical hypothesis test) allows measuring the strength of association between the terms and topics. The greater the Pearson’s chi-square, the more likely the hypothesis of dependence between terms and topic (Carvalho et al. 2020). Co-occurrences of words are analyzed in such a way as to understand their relationships in the contexts of scientific discourse and to construct vocabularies of co-occurring words that are specific to each semantic class. Through this analysis, we were able to identify the different thematic dimensions discussed in the literature and the topics covered, and the relationships among different clusters. This analysis allowed us to answer the first research question.

In a subsequent step, we used the semi-automatic text analysis to measure the association grade between the topics and publication year variable. Again, a positive difference, and a threshold of significance set at chi-square, indicated that a topic had received greater attention in a particular year. Hence, by looking at the grade of association between year and clusters, we were able to determine the evolution of the topics over time and, thus, answer the second research question.

To strengthen these analyses, we also studied the keywords authors had listed in the articles. The keywords analysis helped identify the most studied issues related to food security and geographic focuses.

Finally, we investigated the articles published in Scopus between January 2021 and July 11, 2021 (the last search conducted) using the same procedure applied to the main corpus. After the cleaning procedure, the 2021 database consisted of 2,533 articles (out of 3,672 documents resulting from the search). As for the main corpus, we performed the analysis using both Microsoft Excel and Iramuteq software. However, in this case, the application of Reinert’s (1983) method did not result in a statistically significant analysis, as the percentage of text segments retained (69.16%) was lower than the minimum retention indicated by the literature (70*–*75%). Still, it was possible to use the analysis to identify new topics and trends in the literature on food security after the COVID-19 pandemic. Furthermore, the analysis of the keywords complemented this analysis and allowed us to answer the third research question.

Due to the high number of articles included in the dataset, this review did not aim to investigate the specific content of articles. Instead, it aimed to identify the main trends in the literature and the topics that constitute the core of the theoretical and methodological debate around food security.

## Publications over time and across scientific areas

The present section describes some characteristics of the dataset analyzed, namely the corpus dimension, science areas, and journals most interested in food security, as well as how food security articles published between 2000 and 2020 developed over time.

Table 1 shows a first analysis of the database on food security. Between 2000 and 2020, 21,574 articles were published in English in 3,817 different journals. The high number of both articles and journals is representative of the attention that this topic has received over the last two decades and the multiple angles adopted for its analysis. The multidisciplinary of the literature is also evident in the distribution among different scientific areas. Agricultural and biological science (ABS) is the most prominent subject area, closely followed by social sciences (SS) and environmental science (ES). An important percentage of articles also deals with medicine and health (MH) and economics (E). Therefore, we can affirm that the issue of food security cuts across disciplinary boundaries. Authors have analyzed the issue from a variety of theoretical and methodological perspectives.

**Table 1:** Publications, Science Area, and countries (2000–2020)

Search term	No. of Articles	No. of Journals	Science Area	%	Countries	No. of Articles
“Food security”	21,574	3,817	*Agricultural and Biological Sciences* *Social Sciences* *Environmental Science* *Medicine and Health* *Economics*	21.7 17.5 16.6 8.1 5.1	USA UK CHINA AU INDIA	6,363 2,505 2,327 1,550 461

It is also worth noting that geographically, the publications are mainly concentrated in the Anglo-Saxon countries, with U.S. academia producing almost 30% of the literature. Other than the obvious issue of the English language, the strong preponderance of articles published by British and U.S. universities can be linked to the long tradition of these countries in food security and food assistance programs and evaluation. The automatic analyses performed by the Scopus website allowed us to identify the most relevant scientific areas explored in each country. Both the USA and UK distribute their production rather evenly among different scientific areas (USA: 18.3% ABS, 17.7% SS, 14.9% ES, 13.3% MH; UK: 21.4% ABS, 18.9% ES, 18.5% SS).

China represents an exception to the English-speaking countries, being the third most prolific producer. China has seen a rapid increase in articles published since 2009, with more than half of the studies focusing on ES and ABS. This timing in Chinese scientific production may be due both to the first major food security policy document released by the Chinese central government in 2004 to combat food poverty (Ghose 2014) and the effects of the global food crisis after the global increase in food prices in 2007. The instability was due to smaller amounts of food being available for human consumption because farmers devoted more of their crops to biofuel production in the USA and Europe (Bohstedt 2016).

**Fig. 1:**
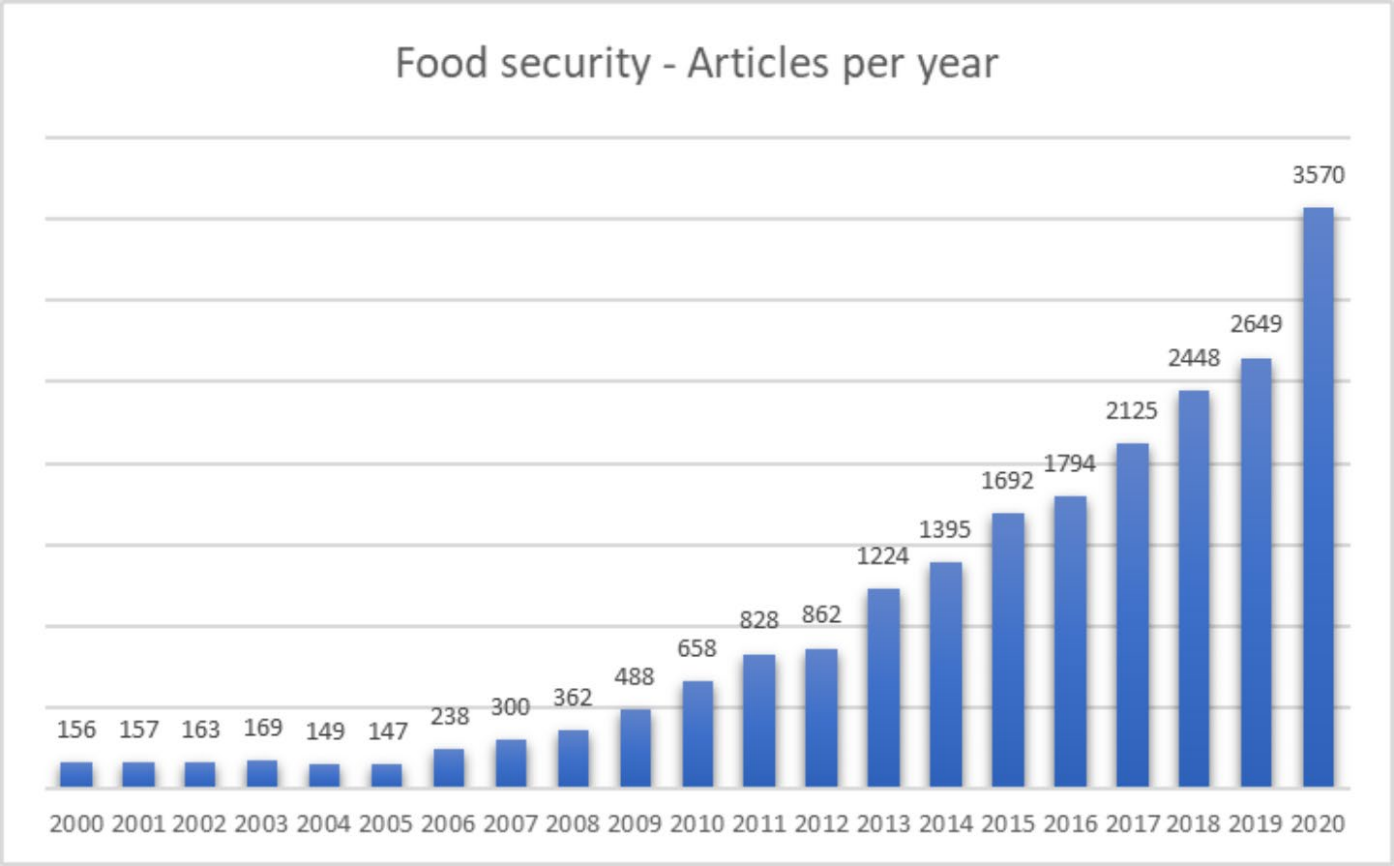
Articles on food security published per year (2000–2020)

**Fig. 2:**
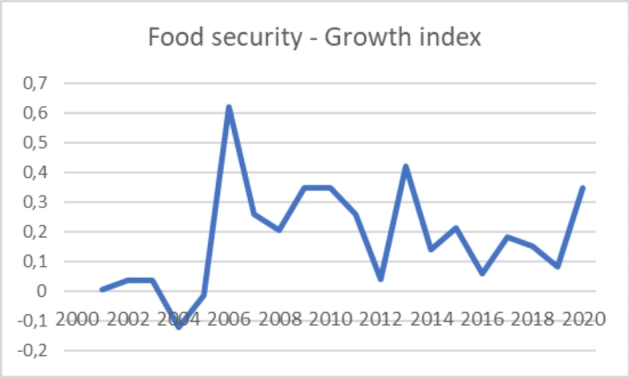
Yearly growth index of articles on food security (2000–2020)

As shown in Fig. 1, the number of articles published every year has strongly and steadily increased since the beginning of the century, with rapid growth in the last decade. The number of publications grew from 156 to 2,000 to 658 in 2010 to over 3,500 in 2020. The growth index represented in Fig. 2 (calculated as [(PresentValue – Past Value)/PastValue] *100) shows more clearly a substantial increase in 2006 (global food crisis) and two peaks in 2013 (effects of the second financial crisis) and 2020 (COVID-19 crisis). These figures show how the food security issue has been gaining importance in the literature over time, a trend confirmed by the number of articles published in 2021. In addition, they confirm the critical link between economic and social crises and food security. These crises increase food insecurity, thus sparking new debates and studies on the issue of food security.

The attention to food security observed in Fig. 2 gained momentum due to a sharp rise in alimentary prices in 2006 and 2013. The first rise was caused by global financial speculation in agricultural commodities futures (McKeon 2014). Moreover, the food crisis that broke out between 2007 and 2008 focused the interest of a broad international scientific debate on agricultural policies, the gaps between the north and the southern countries, and the adequacy of global food security governance’s primary tool: food aids. The second momentum of interest for food insecurity was in 2013. It converges with the new rise in food prices and a growing interest in the social movements and civil society organizations’ role in mitigating the social and health consequences of financial speculation and global food market dynamics on local communities. Finally, from 2019 onwards, the scientific literature has increased interest in the connections between the unstoppable growth of food prices, climate change, and the COVID-19 pandemic on food security. The scientific articles concern, among the others, the challenges posed by climate change on the resilience of local agricultural systems, and finally, the negative impact of policies to combat the COVID-19 pandemic on poverty and the growing fragility of individuals and families in accessing quality food both in developed and developing countries and regions.

As observed in Section 2, the issue of food security results in many different journals and from various perspectives. The plurality of approaches to the problem is also evident in ranking the journals that have published the most articles. As we can see in Table 2, the ten most active journals publish literature which is either interdisciplinary or pertaining to different scientific areas. It is interesting to observe that the first journal is *Sustainability*, thus reflecting the strong link between the issue of food security and sustainability, in particular with regard to the environmental and social aspects of the latter. In addition, food security has a dedicated journal, thus confirming once again its importance in the academic world.

**Table 2:** Distribution of articles on food security (2000–2020) per journal and scientific area

Journal	Scientific area	% Articles per journal	
*Sustainability*	Interdisciplinary (Social Sciences, Environmental Science)		2.25
*Food Security*	Interdisciplinary (Social Sciences, Agricultural and Biological Science)		2.25
*PLoS ONE*	Interdisciplinary (Science, Medicine and Health, Social Sciences)		1.31
*Food Policy*	Interdisciplinary (Social Sciences, Environmental Science, Agricultural and Biological Science)		1.27
*Journal of Hunger and Environmental Nutrition*	Medicine and Health, Social Sciences (Health)		0.99
*Science of the Total Environment*	Environmental Science		0.82
*Public Health Nutrition*	Medicine and Health		0.80
*Land Use Policy*	Interdisciplinary (Social Sciences, Environmental Science, Agricultural and Biological Science)		0.77
*International Journal of Environmental Research and Public Health*	Medicine and Health, Environmental Science		0.70
*Remote Sensing*	Science and Technology		0.70

## Food security thematic dimensions

After mapping publications on food security, we can observe the empirical data that allow us to answer the first research question:

RQ1: What are the main thematic dimensions linked to the theme of food security?

A first attempt to identify the most recurrent issues analyzed by the food security literature can be made by looking at the keywords proposed by the authors, which outline the focus of the articles. As we can see from Table 3, the focus proposed by the keywords is rather composite. The presence of climate change as the second most frequently used keyword is significant of the importance that adaptation (n. 13), resilience to climate change (n. 15), and the sustainability (n. 6) of food systems play in the academic discourse. Food production, represented by words such as agriculture (n. 4), rice (n. 11), and maize (n. 16), is also an important element of food security. It is also important to note the countries that appear as keywords and, thus, represent the main geographical focuses of the literature. The African continent is represented by three keywords: Africa (n. 8), sub-Saharan Africa (n. 12), and Ethiopia (n. 14). The strong presence of Africa in the literature can be linked to both the problem of food security in the continent and the FAO’s strong focus on the region. It is also interesting to observe that China (n.9) is the first country to appear as a keyword. This finding is in line with the fact that China is among the most prolific publishers and among the most studied countries, alongside Africa and India.

**Table 3:** Most frequent authors’ keywords in articles on food security (2000–2020)

	Keywords	Frequency	
1	*food_security*	6,017	
2	*climate_change*	1,194	
3	*food_insecurity*	1,047	
4	*agriculture*	840	
5	*nutrition*	489	
6	*sustainability*	460	
7	*poverty*	428	
8	*Africa*	334	
9	*China*	324	
10	*livelihood*	269	
11	*rice*	266	
12	*sub_Saharan_Africa*	254	
13	*adaptation*	252	
14	*Ethiopia*	250	
15	*resilience*	233	
16	*maize*	233	
17	*India*	230	
18	*gender*	227	
19	*drought*	225	
20	*sustainable_development*	211	

**Fig. 3:**
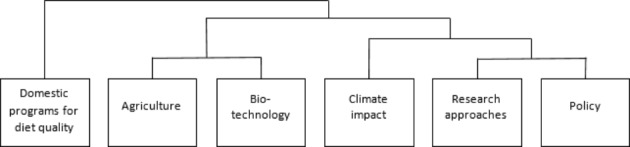
Descending hierarchy of thematic clusters in the food security literature (2000–2020)

After a first analysis of the most discussed topics in the literature, we proceed to the analysis of the thematic clusters. The application of Reinert’s (1983) method to the corpus of food security articles resulted in a division into six thematic clusters that represent six different focuses of the literature. As shown in Fig. 3, the division into clusters follows a hierarchical procedure which divides the texts based on their semantic classes, until the homogeneity of the texts makes a further division impossible. Hence, we can observe a first cluster (domestic programs for diet) isolated from a second macro-cluster that unfolds in three separate sub-clusters (agriculture and biotechnology; research approaches and policy; climate impact).

Table 4 shows the percentage of texts that belong to each cluster and the words that best characterize it. The titles were assigned to each cluster by the authors based on the analysis of the content, to simplify their identification.

**Table 4:** Title, percentage, and characterizing words of the thematic clusters describing the food security literature (2000–2020)

Cluster n.	Title	% Of texts	Characterizing words
Cluster 1	Domestic programs for diet quality	19.5%	Interview, Survey, Questionnaire, Household food security, Obesity, Low income
Cluster 4	Climate impact	18.1%	Food security, Agriculture, Food production, Climate change, Energy
Cluster 6	Policy	17.92%	Strategy, Adaptation, Promote, Tool, Implementation, Capacity, Participatory
Cluster 2	Agriculture	16.38%	Yield, Temperature, Season, Crop, Rice, Rainfall
Cluster 5	Research approaches	15.64%	Concept, Political, Governance, Debate, Food system, Justice, Case studies
Cluster 3	Biotechnology	12.45%	Gene, Plant, Resistance, Tolerance, Stress, Pathogen, Breed

Below is a short description of the content of each cluster and its perspective on food security:

- Cluster 1: “Domestic programs for diet quality” is the biggest thematic cluster in the literature. Moreover, as shown in Fig. 3, this cluster stands as a separate stream of the literature. It deals with food security programs and diet quality, linking food security to household food security. It focuses on programs designed to secure a good dietary intake, with a specific focus on *low-income*^1^ families, gender (*woman, female)*, and *schools*. The American Supplemental Nutrition Assistance Program (SNAP) receives considerable attention within this cluster, as one of the oldest and broadest national food security programs. Hence, food security is analyzed in its dimension related to nutritional quality (*nutritional status, dietary diversity)*, observing the impact of different factors (*income, education, age)* on food insecurity at the individual level. The measurement of food insecurity is central in this cluster, as highlighted by words such as *survey, score, questionnaire*, and *interview*. Through these methods, the literature aims to measure the impact of food security programs in contrasting food insecurity and improving the nutritional status of the participants. Due to its size and its separateness from the rest of the literature, we analyze this cluster in greater depth in the next section.
- Cluster 2: “Agriculture” focuses specifically on agricultural production, investigating the influence of different factors on food production. Specifically, it observes the impact of climatic events (*temperature, season, rainfall*) and agricultural practices (*fertilizers, manure, experiment)* on the *yield* productivity of different *crops* (*maize, wheat, rice, grains).* The approach to food security in this cluster is purely scientific, and it aims to increase and improve food production to guarantee access to food.
- Cluster 3: “Bio-technology” is closely related to Cluster 4, as it deals with the *genetic* and biological aspects of food and its production. The analysis focuses on the genetic modification of *plants* and *species* to improve their *resistance* and *tolerance* to *pathogens* and external *stress.* The approach is purely scientific, and it focuses on improving the resistance of food production to the impact of climate change.
- Cluster 4: “Climate impact” relates to a dimension of *global demand* for *food production*, mostly related to the impact of *climate change* and *population growth*. *Agricultural production* is essential for ensuring food security and global access to food. However, it is facing challenges on two fronts. On the one hand, it needs to deal with the impact of climate change and how it *threatens productivity, biodiversity*, and *water* resources. On the other, it is challenged by changes in *land use*, both under the *pressure* of *urbanization* and about land conversion for producing *energy*, in the form of *biofuel.* Thus, this cluster focuses on improving food security through the adoption of strategies that mainly aim to strengthen the resilience, productivity, and sustainability of food production.
- Cluster 5: “Research approaches” is a rather composite cluster which includes the different *frameworks* and *perspectives* adopted by the food security literature. The approaches and methodologies are very diverse, ranging from *political discussions* and *debates (agenda, discourse)*, to *social* and *justice* perspectives (*movement, human rights)*, to *food systems* and *food sovereignty* (*governance).* It uses both empirical *case studies* and *theoretical* approaches (*theory)* to discuss the *issue* and *concept* of food security. The diversity of approaches reflects the multidisciplinarity of the literature, as previously observed.
- Cluster 6: “Policy” deals with the *strategies* and *policies* that *governments* adopt for food security and *adaptation*. Agricultural policies are central in this cluster, with natural resources and land *management* as essential elements of adaptation strategies and *farmers’ support.* The cluster analyses all phases involved in the policy process, examining the definition of an *agenda*, the *decision-making* process, the *planning* of a *strategy*, the identification of *tools*, and the *adoption* phase, which includes the *implementation, strengthening*, and *building* of *capacities. Participatory* practices, as well as *technology* and *innovation*, receive much attention within the cluster. Thus, this cluster focuses on the improvement of food security through the adoption of strategies that mainly aim at strengthening the resilience, productivity, and sustainability of food production.

It is interesting to observe the role “poverty” plays in the food security literature. While it is one of the most used keywords listed by authors, it does not appear as a frequent word in any clusters (Cluster 2 is the one in which it first appears, n. 223). This difference in importance could be that while poverty is considered a central issue in the food security literature, the articles focus on the causes, effects, and different dimensions of poverty, rather than on poverty itself. Causes and effects are treated in different clusters within the literature. Cluster 2 deals with climate change’s impact on the increasing number of people falling into poverty globally. Cluster 6 and, to a lesser extent, Cluster 5 deal with its effects through programs that aim to alleviate food and nutrition poverty.

## Evolution over time and relation between thematic dimensions

After the analysis of the thematic clusters and their evolution over time, we can answer the second research question:

RQ2: How do these dimensions evolve, and how do they relate?

**Fig. 4:**
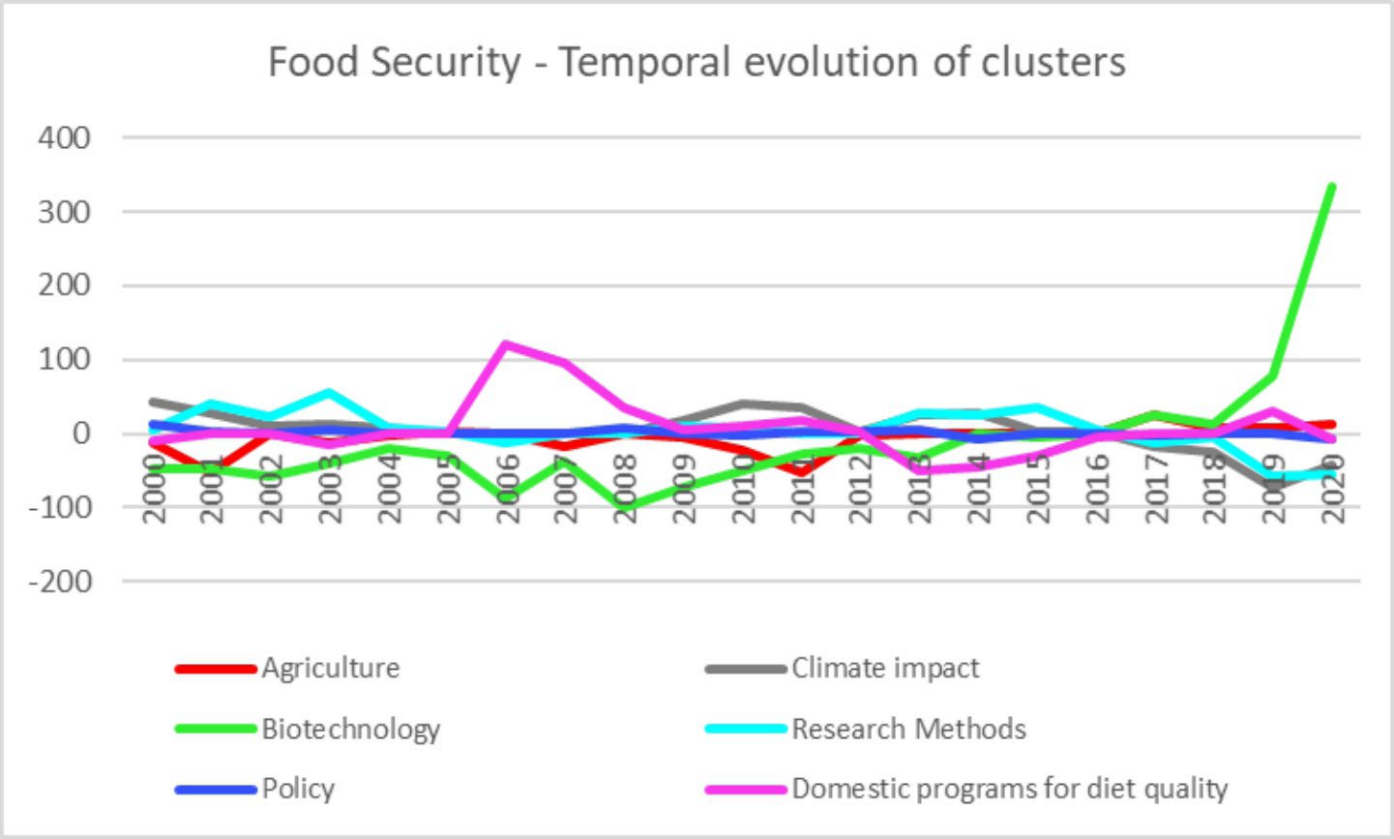
Evolution of thematic clusters on food security by year (y-axis represents the degree of association between topics and years using Pearson’s chi-square test)^2^

Figure 4 shows the evolution over time of the different thematic clusters. We can see a relatively stable trend in the evolution of the clusters.

*Figure compiled by authors*.

Still, we can observe how the “biotechnology” cluster has started to receive increased attention in the past five years, with a growing trend. Likewise, the “domestic programs for diet quality” cluster saw a spike in attention between 2006 and 2007. This increase is likely to be linked to the global food crisis caused by a shift in land use, from food production for human consumption to biomass production for biofuel. The drop-in food production caused a spike in the prices and a consequent increase in food insecurity, thus putting programs to improve food security high on the policy and scholarly agenda.

The food security literature has seen multiple topics co-exist in the last two decades. The “policy” cluster has had the most stable presence in the literature, while other clusters have seen fluctuating trends. It is worth noting that the “diet quality” cluster received considerable attention in 2006**–**2007 and that a growing strand of literature focuses on the technological and biological aspects of food production for food security.

**Fig. 5:**
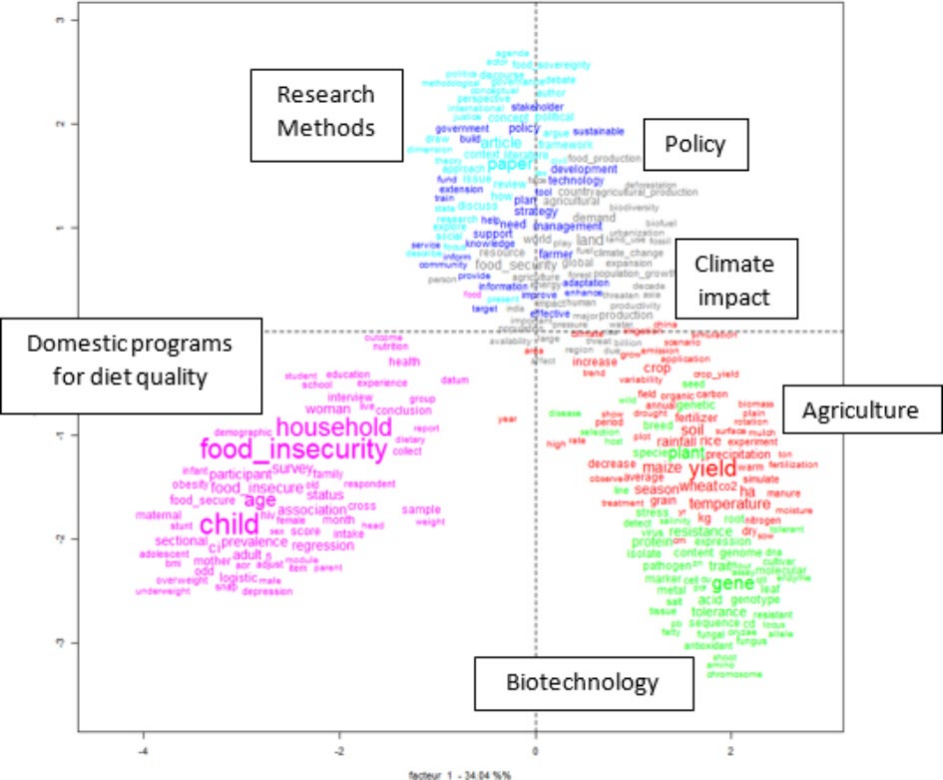
Distance and interconnections between thematic clusters related to food security (2000–2020)

The Cartesian planes (Fig. 5) reflect the relationships between different clusters and among the most frequent words belonging to the clusters themselves. The zero value on the X-axis allows for distinguishing between topics that show a positive correlation among terms and topics that tend to be more isolated. Clusters with a higher co-occurrence appear graphically close, while graphically distant clusters are treated as separate issues in the literature. Moreover, words used most frequently within a cluster appear more prominent in the graph.

As already observed in the descending hierarchy of the cluster (Fig. 3), the six clusters can be grouped into three macro-clusters. The cluster “diet quality programs” stands alone, with little integration with the other clusters. The “agriculture” and “biotechnology” clusters form a macro-cluster dealing with food production. The articles about this macro-cluster focus on improving agricultural production, increasing its resistance to the changing climate, and guaranteeing food security in a world with a fast-growing population. The third macro-cluster includes the methodological aspects and combines the thematic dimension with the impact of global warming. The articles in the macro-cluster, then, focus on the policies that have been implemented to face the challenges proposed by climate change, mainly concerning land management and strategies to prevent and adapt to climate change. The second and third macro-clusters show similarities, but while the second one adopts a techno-biological perspective on the issue, the third employs a socioeconomic perspective.

Hence, the thematic dimensions that can be identified in the food security literature comprise (a) domestic programs to tackle food insecurity; (b) policies to fight the challenges posed to global food security by climate change; and (c) analysis and technological improvements in food production to guarantee global food security.

## Domestic programs for diet quality: an in-depth analysis

It is interesting to observe Cluster 6 in greater depth, as it represents a consistent part of the literature and a stand-alone cluster. To better understand its content, we applied Reinert’s (1983) analysis method to its sub-corpus of text segments. The analysis showed a division into five clusters, two dedicated to methodologies and three dedicated to an analytical dimension.

The methodological clusters show a clear division between qualitative methods, which focus on nutrition security (i.e., dietary diversity) at the individual level, and quantitative ones, which deal with food security (i.e., access to food and poverty) at a more macro level. The qualitative methods, which represent the majority, present a solid link to interviews and participatory approaches and a temporal dimension (name of months), highlighting that attention is given to developing countries and the impact of seasonal cycles on agricultural production. Conversely, the quantitative methods investigate the statistical correlation among different phenomena (e.g., food security and obesity) at the macro, and national level.

The analytical clusters unfold into two different streams in the literature: one develops methodological strategies and techniques, and the other devises in-depth substantive analyses of the issue of food security. The first stream of articles analyses how to measure food security and which instruments to assess its causes and effects ought to be used, including the comparative approach. The second stream adopts a policy approach that aims to answer how to intervene by considering food insecurity programs organizational and institutional contexts. Intervention strategies are defined by analyzing of food security’s risk factors, which can be semantically attributed to a health dimension (i.e., obesity, diseases, and mental health), and a behavioral and social dimension (i.e., purchasing habits, income, and education). These two dimensions are discussed together within the literature, showing how they are intrinsically connected in determining food security.

The relevance of the methodological dimension and the attention to risk factors confirm the importance of developing appropriate tools to measure food security, both in terms of causes and effects, to design more appropriate food security programs.

## New thematic dimensions in the aftermath of COVID-19

The global COVID-19 pandemic has had a consistent impact, from a socioeconomic standpoint and on academic production. Consequently, the COVID-19 impacts (direct and indirect) on the global and local scale increased scholars’ engagement and attention. Hence, it is interesting to analyze the 2021 literature on food security to observe whether the crisis has impacted academic production on the issue and new thematic dimensions have emerged.

Thus, we can answer our third research question:

RQ 3: What new dimensions of the issue do the literature highlight concerning the COVID-19 pandemic?

The articles published in 2021 provide us insights into the COVID-19 impact on the issue of food security and whether the pandemic caused a change in focus in the literature.

In the first six months of 2021, 2,533 food security articles were published in 977 journals. The 2021 data also confirm the saliency of the topic and its multidisciplinary. However, while ABS remains the central area of focus (21.9%), we observed increased attention paid to ES (19%) and a decrease in focus on SS (13.8%). This result is in line with the growing trend in the cluster that deals with biotechnological aspects of food security and emerged by the journals publishing the highest number of articles on food security in 2021. Four out of five of the most active journals deal with ES (*Sustainability*, *International Journal of Environmental Research and Public Health*, *Science of the Total Environment*, and *Journal of Cleaner Production*). However, it is more likely that these trends are linked to the increased scholarly and public attention paid to climate change. In addition, China has confirmed its importance as a publisher of food security literature.

COVID-19 appears as the fourth most frequent keyword proposed by the authors, after food security, food insecurity, and climate change. This result shows how COVID-19 has become the focus of many articles. Still, climate change remains a more substantial concern for academics dealing with food security, as highlighted by the subject areas and journals.

Reinert’s (1983) analysis method can better enlighten the impact of COVID-19 on the literature, showing how the topic is treated in articles and how the clusters have changed in the aftermath of the pandemic. Although the disappearance of the methodological cluster, the thematic clusters of 2021 present many similarities with those of the period 2000**–**2020. However, we observe significant differences in topics within the “policy” and “climate impact” clusters. While the 2000**–**2020 “policy” cluster focused on support policies for agricultural adaptation to climate change, the 2021 “policy” cluster has a stronger focus on sustainability policies, with governments seeming to have more importance and the social and environmental dimension being more directly debated. In addition, the 2000**–**2020 “climate impact” cluster became broader in 2021, including different global challenges.

On the one hand, we saw attention paid to the COVID-19 pandemic. On the other, we saw a switch in attention from agriculture to fishery, as an essential source of food for the global population that is currently under threat due to climate change and unsustainable fishing practices. In addition, a warming planet has disrupted and depleted fisheries worldwide, drawing scientific attention to overfishing as a factor increasing the vulnerability of fisheries.

In terms of relations among the different thematic clusters, we can observe that the “bio-technology” cluster has become more separate from the “agriculture” cluster, while the latter has become more closely linked to the “policy” and “climate impact” clusters. The closer link between food production and the global political dimension may be due to the increased public and political attention paid to the issue of climate change, as well as the growing impact of extreme climate events on food production.

Moreover, we observed that the “domestic programs for diet quality” cluster remains an isolated theme in the literature, confirming the silo approach to food insecurity on a domestic scale and the global issue of food production for a growing population and in a warming planet.

## Discussion and conclusions

The systematic literature analysis addressed by the current article increases our knowledge of the vast and multidisciplinary food security scientific production and its theoretical and research dimensions and trends during the last twenty years. Furthermore, it traces the main lines of scientific interest in the topic and indicates the most transversal issues, weaknesses, and opportunities for future development.

In the period considered, food security becomes increasingly relevant in conjunction with and due to the impact of the economic and financial crises and climate change on the weakest segments of the population, both in developing and rich countries.

The main literature trends highlighted by our research can be summarized as follows:

- The number of articles published has increased enormously in the last two decades, with peaks in 2006, 2013, and 2020, all linked to the outbreak of financial and social crises and their effects on agricultural commodities prices and food system dynamics. The place of the issue of food security within the literature has grown in importance over time. This is because it has become sensitive to the increasingly frequent global economic and health crises (Dodds et al. 2020; Galanakis 2020; O’Hara and Toussaint 2021) and the local environmental impact of climate change (Bohle et al. 1994; Lang et al. 2009).
- Domestic programs for diet quality are the most explored dimension of food security and stand as a separate branch of the literature, considerably ahead of the most discussed macro-dimension of food production, which addresses policies, climate impact, and agriculture.
- Despite a relatively stable trend in the evolution of the main topics detected, we can observe how the “biotechnology” cluster has started to receive increased attention in the past five years.
- The general scientific attention to the “diet quality” issue was highly concentrated in 2006–2007, along with a growing strand of literature focused on the technological and biological aspect of food production for food security.
- The household food security measurement and indicators are the most important scientific topics and, at the same time, separate from other arguments addressed by scholars. This separation indicates the difficulty of theoretically and empirically linking the micro nutritional aspects of food security to meso and macro elements, such as government agricultural policy and climatic impact on local diet change. Thus, even though food security is widely recognized as a multidimensional and cross-sectoral issue, academic analyses still seem affected by silo approaches in one of the most important fields of scientific analysis and public intervention.
- The analysis of the articles published in 2021 confirms that ABS remains the central area of focus; increased scholarly and public attention to climate change impact on agriculture production and fishing has emerged in 2021.

Our analysis suggests some conclusive reflections concerning the need to link more solidly micro studies on food safety and nutrition poverty to macro changes such as climate change impact on agricultural production. However, the potential inherent in the extensive and multidisciplinary research on food safety has limitations, particularly the difficulty of connecting theoretically and empirically the global and regional dimensions of change (crisis) with the meso (policy) and micro (individual behavior) dimensions. Nevertheless, this greater connection can only benefit the action of governments and policies that have long been committed to this front.

## Footnotes

1 The words that belong to the cluster are written in italics.

2 Refer to Sect. 2 for the general meaning of the chi-square value. In the temporal evolution of topics, Pearson’s chi-square indicates the association between the term “year_year of publication” (e.g., “year_2020”) related to each article and the topics. A positive value in the graph suggests over-representation of a topic in a specific year (highly discussed in the literature), while negative values indicate under-representation of the topic in that year (seldomly discussed in the literature)

## References

[CR1] Ahn S, Norwood FB (2020). Measuring Food Insecurity during the Covid-19 Pandemic of Spring 2020. Appl. Economic Perspect. Policy.

[CR2] Arouna A, Soullier G, Mendez del Villar P, Demont M (2020). Policy Options for Mitigating Impacts of COVID-19 on Domestic Rice Value Chains and Food Security in West Africa. Global Food Security.

[CR3] Béné C (2020). Resilience of Local Food Systems and Links to Food Security–A Review of Some Important Concepts in the Context of COVID-19 and Other Shocks. Food Secur..

[CR4] Besley T, Kanbur R (1988). Food Subsidies and Poverty Alleviation. Econ. J..

[CR5] Bohle HG, Downing TE, Watts MJ (1994). Climate Change and Social Vulnerability: Toward a Sociology and Geography of Food Insecurity. Glob. Environ. Change.

[CR6] Bohstedt J (2016). Food Riots and the Politics of Provisions from Early Modern Europe and China to the Food Crisis of 2008. J. Peasant Stud..

[CR7] Burgess R, Stern N, Ahmad E, Dreze J, Hills J, Sen A (1991). Social Security in Developing Countries: What, Why, Who, and How?. Social Security in Developing Countries. WIDER studies in development economics.

[CR8] Cable, J., Jaykus, L., Hoelzer, K., Newton, J., Torero, M.: The Impact of COVID-19 on Food Systems, Safety, and Security—a Symposium Report. Annals of the New York Academy of Sciences 1484(1), 3–8 (2021). 10.1111/nyas.14482 10.1111/nyas.14482 32860255

[CR9] Cafiero C, Viviani S, Nord M (2018). Food Security Measurement in a Global Context: The Food Insecurity Experience Scale. Measurement.

[CR10] Candel JL (2014). Food Security Governance: A Systematic Literature Review. Food Secur..

[CR11] Carlson SJ, Andrews MS, Bickel GW (1999). Measuring Food Insecurity and Hunger in the United States: Development of a National Benchmark Measure and Prevalence Estimates. J. Nutr..

[CR12] Carvalho TS, Mota DM, Saab F (2020). Utilização do software IRaMuTeQ na análise de contribuições da sociedade em processo regulatório conduzido pela Agência Nacional de Vigilância Sanitária. Vigilancia Sanitaria Em Debate.

[CR13] Chan J, To H, Chan E (2006). Reconsidering Social Cohesion: Developing a Definition and Analytical Framework for Empirical Research. Soc. Indic. Res..

[CR14] Dodds K, Broto VC, Detterbeck K, Jones M, Mamadouh V, Ramutsindela M, Varsanyi M, Wachsmuth D, Woon CY (2020). The COVID-19 Pandemic: Territorial, Political and Governance Dimensions of the Crisis. Territory, Politics, Governance.

[CR15] Dowler EA, O’Connor D (2012). Rights-Based Approaches to Addressing Food Poverty and Food Insecurity in Ireland and UK. Soc. Sci. Med..

[CR16] El-Taliawi OG, Goyal N, Howlett M (2021). Holding out the promise of Lasswell’s dream: Big data analytics in public policy research and teaching. Rev. Policy Res..

[CR301] Esobi, I. C. et al.: Food Insecurity, Social Vulnerability, and the Impact of COVID-19 on Population Dependent on Public Assistance/SNAP: A Case Study of South Carolina. USA. J. Food Secur. **9**(1), 8?18 (2021)

[CR17] Galanakis CM (2020). The Food Systems in the Era of the Coronavirus (COVID-19) Pandemic Crisis. Foods.

[CR18] Ghose B (2014). Food Security and Food Self-Sufficiency in China: From Past to 2050. Food Energy Secur..

[CR19] Haddad, L.J., Peña, C., Nishida, C., Quisumbing, A.R., Slack, A.: Food Security and Nutrition Implications of Intrahousehold Bias: A Review of Literature. International Food Policy Research Institute (IFPRI), Food Consumption and Nutrition Division. FCND Discussion Paper Number 19. Washington DC. (1996)

[CR20] Janssen MA (2007). An Update on the Scholarly Networks on Resilience, Vulnerability, and Adaptation within the Human Dimensions of Global Environmental Change. Ecol. Soc..

[CR21] Janssen MA, Ostrom E (2006). Resilience, Vulnerability, and Adaptation: A Cross-Cutting Theme of the International Human Dimensions Program on Global Environmental Change. Glob. Environ. Change.

[CR22] Janssen MA, Schoon ML, Ke W, Börner K (2006). Scholarly Networks on Resilience, Vulnerability and Adaptation within the Human Dimensions of Global Environmental Change. Glob. Environ. Change.

[CR23] Kendall A, Olson CM, Frongillo Jr EA (1995). Validation of the Radimer/Cornell Measures of Hunger and Food Insecurity. J. Nutr..

[CR24] Lang T, Barling D, Caraher M (2009). Food Policy: Integrating health, environment and society.

[CR25] Leyna GH, Mmbaga EJ, Mnyika KS, Klepp K-I (2008). Validation of the Radimer/Cornell Food Insecurity Measure in Rural Kilimanjaro, Tanzania. Public Health. Nutr..

[CR26] Maxwell S (1996). Food Security: A Post-Modern Perspective. Food policy.

[CR27] Maxwell S, Smith M (1992). Household Food Security: A Conceptual Review.

[CR302] McKeon, N.: Food security governance: Empowering communities, regulating corporations. London, Routledge (2014)

[CR28] Mechlem K (2004). Food Security and the Right to Food in the Discourse of the United Nations. Eur. Law J..

[CR29] Mishra K, Rampal J (2020). The COVID-19 Pandemic and Food Insecurity: A Viewpoint on India. World Dev..

[CR30] Moseley WG, Battersby J (2020). The Vulnerability and Resilience of African Food Systems, Food Security, and Nutrition in the Context of the COVID-19 Pandemic. Afr. Stud. Rev..

[CR303] Nestle, M.: The Supplemental Nutrition Assistance Program (SNAP): History, Politics, and Public Health Implications. Am. J. Public Health **109**(12), 1631?35 (2019) 10.2105/AJPH.2019.305361 PMC6836773 31693415

[CR31] Nosratabadi S, Khazami N, Abdallah MB, Lackner Z, Band S, Mosavi S, Mako C (2020). Social Capital Contributions to Food Security: A Comprehensive Literature Review. Foods.

[CR32] O’Hara S, Toussaint EC (2021). Food Access in Crisis: Food Security and COVID-19. Ecol. Econ..

[CR33] Pranckute R (2021). Web of ˙Science (WoS) and Scopus: The Titans of Bibliographic Information in Today’s Academic World. Publications.

[CR35] Radimer, K.L., Olson, C.M., Greene, J.C., Campbell, C.C., Habicht, J.-P.: Understanding Hunger and Developing Indicators to Assess It in Women and Children. Journal of Nutrition Education 24(1, Supplement 1), 36S-44S doi: (1992). 10.1016/s0022-3182(12)80137-3

[CR36] Radimer KL, Radimer KL (2002). Measurement of Household Food Security in the USA and Other Industrialised Countries. Public Health. Nutr..

[CR37] Reinert M (1983). Une méthode de classification descendante hiérarchique: application à l’analyse lexicale par contexte. Cahiers de l’analyse des données.

[CR38] Reinert, M.: Alceste Une Méthodologie d’analyse Des Données Textuelles et Une Application: Aurelia De Gerard De Nerval. Bulletin of Sociological Methodology/Bulletin de méthodologie sociologique 26(1), 24–54 (1990)

[CR39] Reinert, M.: Alceste Une Méthode Statistique et Sémiotique d’analyse de Discours. Application Aux Rêveries Du Promeneur Solitaire. Revue française de psychiatrie et de psychologie médicale 5(49), 32–36 (2001)

[CR40] Righettini, M.S., Lizzi, R.: How scholars break down “policy coherence”: The impact of sustainable development global agendas on academic literature. Environ. Policy Gov. 1–12 (2021). 10.1002/eet.1966

[CR41] Sbalchiero S, Tuzzi A (2018). Topic Detection: A Statistical Model and a Quali-Quantitative Method. Tracing the Life Cycle of Ideas in the Humanities and Social Sciences.

[CR42] Sbalchiero S, Eder M (2020). Topic Modeling, Long Texts and the Best Number of Topics. Some Probl. Solutions Qual. Quantity.

[CR43] Sen, A., World Bank: Levels of Poverty: Policy and Change. Washington, DC,. World Bank Staff Working Paper No. 401. (1980)

[CR44] Sen, A., Issues in the Measurement of Poverty. In: Measurement in Public Choice, Springer, 144–66 (1981)

[CR45] Sen, A., The Food Problem: Theory and Policy. Third World Quarterly 4(3), 447–59: (1982)

[CR46] Smith M, Pointing J, Maxwell S (1993). Household Food Security: Concepts and Definitions: An Annotated Bibliography.

[CR304] Swann, C. A.: Household History, SNAP Participation, and Food Insecurity. Food Policy **73**, 1?9 (2017)

[CR47] Thompson HE, Berrang-Ford L, Ford JD (2010). Climate Change and Food Security in Sub-Saharan Africa: A Systematic Literature. Rev. Sustain..

[CR48] Wekerle GR (2004). Food Justice Movements: Policy, Planning, and Networks. J. Plann. Educ. Res..

[CR49] Zerafati Shoae N, Omidvar N, Ghazi-Tabatabaie M, Houshiar Rad A, Fallah H, Mehrabi Y (2007). Is the Adapted Radimer/Cornell Questionnaire Valid to Measure Food Insecurity of Urban Households in Tehran. Iran? Public. Health Nutrition.

